# Evaluation of compliance and outcomes of a management protocol for massive postpartum hemorrhage at a tertiary care hospital in Pakistan

**DOI:** 10.1186/1471-2393-11-28

**Published:** 2011-04-13

**Authors:** Lumaan Sheikh, Nida Najmi, Umair Khalid, Taimur Saleem

**Affiliations:** 1 Department of Gynecology and Obstetrics, Aga Khan University, Stadium Road, Karachi 74800, Pakistan; 2 Medical College, Aga Khan University, Aga Khan University, Stadium Road, Karachi 74800, Pakistan

## Abstract

**Background:**

Massive postpartum hemorrhage is a life threatening obstetric emergency. In order to prevent the complications associated with this condition, an organized and step-wise management protocol should be immediately initiated.

**Methods:**

An evidence based management protocol for massive postpartum hemorrhage was implemented at Aga Khan University Hospital, Karachi, Pakistan after an audit in 2005. We sought to evaluate the compliance and outcomes associated with this management protocol 3 years after its implementation. A review of all deliveries with massive primary postpartum hemorrhage (blood loss ≥ 1500 ml) between January, 2008 to December, 2008 was carried out. Information regarding mortality, mode of delivery, possible cause of postpartum hemorrhage and medical or surgical intervention was collected. The estimation of blood loss was made via subjective and objective assessment.

**Results:**

During 2008, massive postpartum hemorrhage occurred in 0.64% cases (26/4,052). No deaths were reported. The mean blood loss was 2431 ± 1817 ml (range: 1500 - 9000 ml). Emergency cesarean section was the most common mode of delivery (13/26; 50%) while uterine atony was the most common cause of massive postpartum hemorrhage (14/26; 54%). B-lynch suture (24%) and balloon tamponade (60%) were used more commonly as compared to our previously reported experience. Cesarean hysterectomy was performed in 3 cases (12%) for control of massive postpartum hemorrhage. More than 80% compliance was observed in 8 out of 10 steps of the management protocol. Initiation of blood transfusion at 1500 ml blood loss (89%) and overall documentation of management (92%) were favorably observed in most cases.

**Conclusion:**

This report details our experience with the practical implementation of a management protocol for massive postpartum hemorrhage at a tertiary care hospital in a developing country. With the exception of arterial embolization, relatively newer, simpler and potentially safer techniques are now being employed for the management of massive postpartum hemorrhage at our institution. Particular attention should be paid to the documentation of the management steps while ensuring a stricter adherence to the formulated protocols and guidelines in order to further ameliorate patient outcomes in emergency obstetrical practice. More audits like the one we performed are important to recognize and rectify any deficiencies in obstetrical practice in developing countries. Dissemination of the same is pivotal to enable an open discourse on the improvement of existing obstetrical strategies.

## Background

Primary postpartum hemorrhage (PPH) is the leading cause of maternal morbidity and mortality especially in developing countries where the availability of safe cesarean procedures is not universal [1]. The incidence of primary PPH (blood loss ≥ 500 ml) at our institution has been previously reported to be around 2.9% of all vaginal deliveries [2].

Loss of 500 ml of blood in the first 24 hours following delivery is generally considered as physiologically normal, and anything exceeding that constitutes PPH. Significant clinical deterioration usually does not occur until there is a blood loss of > 1000 - 1500 ml [3,4]. "Massive" primary PPH occurs when there has been an estimated blood loss of ≥ 1500 ml, peri-partum fall in hemoglobin concentration > 4 g/dl or acute transfusion requirement of 4 units of blood [5]. In our experience at a tertiary care hospital in Pakistan, a developing country; massive PPH was reported in 0.5% cases out of a total 4881 deliveries [2].

Massive primary PPH can result in maternal complications like hypovolemic shock, disseminated intravascular coagulation, hepatic dysfunction, adult respiratory distress syndrome and renal failure. In order to prevent these complications, an organized and step-wise management protocol should be immediately initiated. It is important to be cognizant of the fact that despite the growing body of literature on the known risk factors for primary PPH, it maybe inevitable in some cases. Hence, active management of the third stage of labor should be offered to all women. This includes the administration of uterotonic agents, controlled cord traction, and uterine massage after delivery of the placenta [6].

Immediate resuscitation with attempts to treat the cause forms the cornerstone of management of PPH. Evidence-based guidelines for the management of PPH have been formulated in order to provide an organized and standardized plan of care for the different scenarios encountered in clinical practice [7].

We conducted a cross-sectional study in 2005 at a tertiary care centre in Karachi whereby we retrospectively reviewed the patient records to assess the current practices of the management of massive primary PPH in the setting of a developing country. An indigenous protocol was then devised based on the results of this audit to set up a standard of care [see additional file 1] which was applied at our institution. This protocol was implemented by sensitizing the obstetric and other related staff through multiple presentations, drills and simulated scenarios. They were also taught the proper techniques and skills for performing the different steps of the protocol. This current paper aims to evaluate the compliance and impact of our proposed management protocol three years after its introduction at our institution.

## Methods

### Study setting and design

A review of deliveries with massive primary PPH at Aga Khan University Hospital (AKUH), Karachi, Pakistan between January, 2008 to December, 2008 was carried out. AKUH, Karachi, established in 1985 as a part of the Aga Khan Development Network, is a philanthropic teaching tertiary care hospital in Pakistan. This 563-bed hospital has 15 inpatient units including a dedicated Obstetrics and Gynecology unit. The hospital provides health care services to 50,000 inpatients and 600,000 outpatients annually from all over Pakistan. The average length of stay for hospitalized patients is 3.4 days at AKUH, Karachi. The hospital has a total of 19 surgical theatres; 11 of these theatres are the main operating theatres while the remaining theatres are used for procedures done as daycare, in the emergency room or the community health center. The Obstetrics and Gynecology unit of AKUH, Karachi, comprises of 88 beds with 12 bedded labor and delivery suites and a 24 hours obstetric operating service. This is a consultant-led maternity service, staffed with experienced midwives, resident nurses, postgraduate trainees, on floor registrars and specialist registrars. Normal deliveries are conducted by the senior postgraduate trainee. These are defined as spontaneous vaginal deliveries (SVDs) without any complications. Overall, the cesarean section rate at AKUH is 25%. The maternal mortality rate (MMR) in 2008 was 132.9 per 100,000 live births (all unbooked maternal deaths) while the perinatal mortality rate (PMR) for the same time period was 5.84 per 1,000 live births.

Regarding drills and teaching programs for emergency obstetric care, the unit at AKUH is certified by the American College of Family Physicians for Advanced Life Support in Obstetrics (ALSO) since 2002. Since then, ALSO training courses are conducted regularly twice a year for the health care professionals involved in the provision of obstetric care. More generalized but structured teaching activities such as drills and workshops are also conducted for the trainees on a regular basis.

### Inclusion criteria

Only booked deliveries with massive primary PPH were included. Massive primary PPH was defined as blood loss ≥ 1500 ml.

### Estimation of blood loss

The estimation of blood loss was made via subjective as well as objective assessment. Subjective measures included counting of soaked swabs, estimation of blood clots and blood in the suction bottle whereas objective assessment was made via serial measurement of the reduction in hemoglobin level or evaluation of the need for blood transfusions [2]. Active management of the third stage of labour is offered to all women delivering at our institution [2].

### Data management and statistical analysis

We reviewed the charts of all patients who fulfilled our inclusion criteria and gathered data on a structured, pre-tested proforma prepared for the purpose [see additional file 2]. Information regarding mortality, mode of delivery, possible cause of PPH and therapeutic medical or surgical intervention was collected. The data obtained from the file review were coded and entered in SPSS version 16.0 for analysis. Descriptive statistics have been presented for a consecutive series of patients with massive primary PPH.

### Ethical considerations

The protocol of this study was granted exemption from the Ethical Review Committee (ERC) at AKUH because of its retrospective design (as per guidelines of the ERC). Informed consent was not needed owing to the anonymous presentation of the patient data; this was also in accordance with the guidelines of ERC at AKUH.

## Results

Twenty six patients fulfilled the inclusion criteria for this study. During 2008, there were a total of 4052 deliveries at AKUH, Karachi; therefore massive PPH occurred in less than 1% (26/4052) of cases during 2008. No deaths were reported. The mean blood loss was 2431 ± 1817 ml (range: 1500 - 9000 ml).

### Mode of delivery

Among the 26 patients, emergency cesarean section (CS) was the most common mode of delivery (13/26, 50%) followed by spontaneous vaginal delivery with episiotomy (6/26, 23%) and elective CS (3/26, 12%). Forceps delivery with episiotomy and vacuum delivery with episiotomy each occurred in 2 out of 26 cases (8%).

### Cause of PPH

Uterine atony alone was the most common cause of massive PPH in our series (14/26; 54%). Seven patients out of 26 (27%) underwent CS for placenta previa; they experienced massive bleeding from the placental bed after removal of placenta. Out of these seven patients, three had elective CSs and four were operated in emergency due to antepartum hemorrhage secondary to major previa. All the previa were antenatally diagnosed. Two patients out of the seven underwent cesearean hysterectomy to control bleeding. Four patients out of 26 (15%) had retained placenta and underwent manual removal of retained placenta (MROP); atony occurred in these patients after MROP. One patient out of 26 (4%) had trauma in the form of cervical and vaginal tear; this was due to failed vacuum delivery followed by forceps delivery. Bleeding in this patient was eventually controlled by examination under anesthesia (EUA) and suturing. EUA entails a reliable and detailed examination of the cervix, vagina and uterine cavity under anesthesia.

### Compliance with steps of management protocol

The compliance with the steps of management protocol in cases of massive PPH during 2008 at AKUH, Karachi is shown in table 1. It was seen that "call for help", as a step of the management protocol, was followed in all 26 cases (100%). Either a consultant was already present on the spot or was called upon as soon as PPH occurred. Checking vitals of the patient during the management of PPH was not documented in 5 of the 26 cases (19%); all of these cases were managed in the labour room after vaginal delivery. Similarly, the cases where documentation of maintaining two peripheral cannulas and fluids was missing had deliveries in the labour room. Foley's catheter was placed immediately to record urinary output in all 26 cases except one; this patient had PPH secondary to cervical and vaginal tears after failed vacuum delivery followed by instrument assisted delivery. She was later shifted to the operation theatre for EUA. After suturing of the tears, she was catheterized. Performance of uterine massage was not documented in 4 of the 26 cases (15%); two of these patients underwent lower segment CS (LSCS), one had MROP and atony after MROP while one had cervical and vaginal tears following forceps delivery. Placental examination was not done in 9 out of 26 patients (35%). This was either not performed or not possible because of the piece meal removal of morbidly adherent placenta.

**Table 1:** Compliance with steps of management protocol for massive PPH (n = 26)

Step of protocol	Frequency (%)
1. Call for help	26 (100)

2. Check vitals	21 (80.8)

3. Two I/V* cannula	21 (80.8)

4. I/V fluids/colloid	25 (96.2)

5. Cross match blood and labs sent	23 (88.5)

6. Foley's catheterization done	25 (96.2)

7. Uterine massage done	22 (84.6)

8. Genital inspection performed	25 (96.2)

9. Placental inspection done	17 (65.4)

10. EUA and proceed (for trauma/placental removal/hematoma)	6 (23.1)

* I/V - Intravenous

Table 2 shows the general management of patients with massive PPH at our institution. It was seen that overall documentation of management, tailoring recovery phase according to the specific condition of the patient initiation of blood transfusion were the management steps that were most consistently followed overall.

**Table 2:** General management of the patients (n = 26) with PPH

General management steps	Frequency (%)
1. Initiation of blood transfusion at 1500 ml	23 (88.5)

2. Multidisciplinary approach acquired	9 (34.6)

3. Overall documentation of management	24 (92.3)

4. Documentation of counseling after management (patient or family)	18 (69.2)

5. Recovery phase followed according to patient's condition	26 (100)

### Management of uterine atony

A total of 25 out of the 26 cases (96%) had uterine atony with or without association with other conditions; this includes cases that had uterine atony after MROP or severe placental bed bleeding. Table 3 shows compliance with the steps in our institutional protocol with regards to the management of cases with uterine atony. Only 2 out of 26 patients (8%) underwent "examination under anesthesia and proceed". One of these patients had spontaneous vaginal delivery with episiotomy; she was diagnosed as having atony after EUA. The other patient underwent CS and had heavy per vaginal bleeding; she underwent balloon tamponade and vaginal packing under EUA. It was noted that no patient had primary laparotomy for atony; although two patients had intra-abdominal packing done after CS. These patients had re-laparotomy after 48 hours interval for the removal of abdominal packs. Internal iliac vessel ligation was not performed in any patient. Cesarean hysterectomy was done in a total of 3 patients (12%). Two of these patients had LSCS for placenta previa that was morbidly adherent; these patients also had urinary bladder injury during the process which was repaired primarily. The third patient had uterine atony and received hysterectomy.

**Table 3:** Management steps followed in cases of uterine atony (n = 25)

Steps of protocol	Frequency (%)
1. Syntocinon 10 units (upto four doses given)	24 (96.0)

2. Syntocinon infusion (40 units) started	25 (100)

3. Misoprostol (600-800 mcg)	18 (72.0)

4. PGF_2 _alpha (5 mg)	4 (16.0)

5. Balloon tamponade	15 (60.0)

6. EUA done and proceeded	2 (8.0)

7. Uterine/vaginal packing done	8 (32.0)

8. Syntocinon infusion (80 units) started	16 (64.0)

9. Laparotomy for atony performed	0 (0)

10. B- lynch suture applied	6 (24.0)

11. Internal iliac vessel ligation	0 (0)

12. Hysterectomy performed	3 (12.0)

## Discussion

PPH, a leading cause of maternal mortality, causes an estimated 140,000 deaths each year globally - this roughly corresponds to one woman dying every 4 minutes. In addition, serious morbidity may follow PPH in the form of various sequelae [8,9]. Massive PPH is associated with a worse prognosis. Inability to stabilize a patient who is in hemorrhagic shock can eventually result in death [8].

### Incidence of massive PPH

The incidence of massive PPH in this audit was 0.64% of all booked deliveries in 2008 at AKUH, Karachi, Pakistan. This figure was slightly higher but certainly comparable to that reported in our previous experience three years ago (0.5%) [2]. However, it was lower when compared to population-based studies where the incidence of massive PPH has been reported to be as high as 1.1% [10]. In a recent review, PPH was also found to have an increasing trend in the more developed parts of the world including Australia, Canada, the UK and the USA [11]. Although the exact reasons for this increasing trend remain nebulous, the authors of this review identified certain associated factors such as increasing maternal age at childbirth and increase in induced labor, CS and multiple pregnancies [11].

### Mode of delivery

In our previous experience, 56% (18/32) of the women who had massive PPH delivered vaginally. In our more recent experience, the mode of delivery in half of our cases of massive PPH was CS. This association between massive PPH and CS is comparable to other studies, where the highest risk of massive PPH has been reported for pre-labor CS and emergency CS, especially in mothers with previous CS. Hence, caution and vigilance for massive PPH should be exercised with pre-labor and emergency CS [10].

### Causes of PPH

Uterine atony, lower genital tract lacerations, uterine rupture or inversion, retained products of conception and underlying coagulopathy are some of the common causes of PPH [9]. By far the most common etiology is uterine atony - this also emerged as the most common cause of massive PPH in our study. Other causes that we observed include placental adhesion, retained placenta and cervical or vaginal tears. This is comparable to our results three years ago when uterine atony was the most common cause, followed by vaginal haematoma, cervical or vaginal tears, adherent placenta, uterine angle extension and retained placenta [2].

### Management of massive PPH

Timely recognition and intervention are fundamental in preventing serious maternal morbidity and mortality from massive PPH. A combination of conservative therapies is adequate and successful in most cases. However, when the hemorrhagic process continues and when either clotting abnormalities or hemodynamic instability develop, the next step must be an invasive intervention [12].

#### a. Compliance with protocol

In order to improve the management of massive PPH, we proposed a treatment protocol for the management of PPH that has been described earlier [2]. We compared the individual steps of this protocol in terms of compliance. In 8/10 steps, the documented compliance of more than 80% was observed; this is indicative of generally compliant practices at our institution. Our outcomes were improved because no morality occurred during 2008 from massive PPH. In comparison, there was one death (3.1%) in our previous study [2].

#### b. Transfusion of blood products

Traditional blood components, including packed red blood cells, platelets, plasma, and cryoprecipitate, should be used in patients with significant bleeding. Recent studies underline the utility of transfusing these components in defined ratios to prevent dilutional coagulopathy. Disseminated intravascular coagulation (DIC) should be considered in severely bleeding obstetric patients and should be treated aggressively using blood components. About 88% of the patients in our study required blood transfusions. This is higher compared to our experience three years back, when blood transfusions were required in 56% of women who had massive postpartum hemorrhage [2]. This finding can be explained by the fact that adherence to a formulated protocol this time provided a more objective approach to the evaluation for the need of transfusion.

#### c. Recombinant factor VII

Newer hemostatic agents, such as activated factor VII, will play significant roles in patients with bleeding that is refractory to standard therapy [13]. In our series, two patients received recombinant factor VII; the number of doses given was 1 (1.2 mg) and 2 (1.2 × 2 = 2.4 mg) respectively. Hemorrhage was controlled in both patients within 45 to 60 minutes without any adverse effects observed. Both patients had placenta percreta; hysterectomy was indicated in both patients and was then done electively. Post delivery, the bleeding in these patients was controlled via pelvic packing, replacement of clotting factors and ultimately through the use of recombinant factor VII. The patients stabilized after receipt of the latter therapy. The decision to administer factor VII in these patients was made in consultation with the hematologist, anesthesiologist and obstetrician. The intention of giving factor VII in these patients was not the prevention of hysterectomy but rather the control of massive and life threatening PPH. It has been shown in a Pakistan-based study that activated recombinant factor VII can be a life-saving drug in patients with massive PPH [14]. Although implementation of a management protocol for obstetric bleeding that integrates new knowledge in coagulation may aid physicians in improving outcomes for the mother as well as the baby [13], consideration also needs to be given to the ubiquitous availability of these newer modalities.

#### d. Misoprostol

Misoprostol, a prostaglandin analog, is absorbed effectively from rectal as well as oral and vaginal mucosa. Rectally administered misoprostol appears to be an effective treatment for postpartum hemorrhage unresponsive to oxytocin and ergometrine. Given that it is an inexpensive and stable drug, misoprostol has considerable potential to reduce maternal mortality from postpartum hemorrhage in developing countries like Pakistan [15]. Based on the recommendations of a recent meta-analysis [16], a single dose of misoprostol (600 micrograms, oral or sublingual) should be used in instances when other treatments have either failed to work or are not available. A similar study from Pakistan [17] further supports misoprostol's promise as an adjunct treatment option for PPH. We used misoprostol in 72% of our massive PPH cases with good results.

#### e. B-Lynch suture

The B-Lynch surgical technique for the management of massive PPH has been used successfully since 1989. It allows for conservation of the uterus for subsequent menstrual function and pregnancies; hence it is preferred over hysterectomy [18,19]. We managed 24% of our massive PPH cases using B-Lynch sutures, and we strongly recommend this technique based on our successful experience.

#### f. Balloon tamponade

Intrauterine tamponade with balloon catheter is another option that can be employed for the management of massive PPH. A study of patients with severe PPH who were managed with this technique has been reported in literature. Although the tamponade was effective in most cases, 10% of the cases required hysterectomy despite successful placement of the catheter [20]. We used balloon tamponade effectively in 60% of our patients, and it served as a useful adjunct option. In contrast, very few cases employed balloon tamponade (2 cases) and compression sutures (2 cases) in our previous audit [2].

#### g. Arterial embolization

Arterial embolization can be a suitable management option in selected patients with massive PPH, especially in cases where the initial measures have failed and the patient desires fertility. In such patients, an active effort should first be made to investigate potentially correctable causes of massive PPH through trial of uterotonic drugs, the exploration of the uterine cavity, cervix, vagina and inspection of the perineum. Uterine artery embolization can also be considered electively to reduce blood loss in already diagnosed cases of major previas and morbidly adherent placenta. None of our patients with massive PPH were managed via embolization and this treatment option may have been underutilized at our institution. In an experience with 36 cases of severe PPH, arterial embolization was associated with immediate success in all cases. Rare complications from the procedure include puncture site false aneurysm, regressive lower limb paraesthesia, femoral vein thrombosis, and minor puncture site hematomas [21].

### Strengths and limitations

Based on our indigenous experience, we developed a management protocol three years ago for the purpose of improving management of massive PPH. This protocol was designed with special reference to the unique needs and perspectives of developing countries. We have now presented our current experience with its implementation in actual obstetric practice in order to evaluate the feasibility and compliance with this protocol.

This protocol serves as an evidence based model that can reduce practice variability and set up a more uniform standard of care, especially in developing countries. This study is important in terms of describing the trends observed with a management protocol of massive PPH in the setting of a developing country.

We acknowledge that the short duration of the study period and small sample size are the limitations of this study. PPH is one of the top most causes of maternal mortality in a developing country like ours. Larger scale studies are required at different tertiary care hospitals of Pakistan in order to acquire a better and more holistic picture of the impact of this management protocol. Another limitation of this study is that although the compliance of our overall practices was assessed, individual practices of all health care workers involved in the management were not evaluated separately.

## Conclusions

In conclusion, massive PPH is an obstetric emergency that can occur following vaginal delivery or CS. In order to prevent fatal complications, prompt diagnosis and management is imperative in all cases of massive PPH. Three years back, more conventional techniques were opted for at our institution, probably due to less familiarity and experience with the newer and perhaps simpler techniques. After the implementation of this protocol in routine management at our hospital, the latter were more frequently employed with the exception of arterial embolization. It should be emphasized that particular attention needs to be accorded to the documentation of the management steps for better medico-legal practice in developing countries. Additionally, stricter adherence to formulated protocols and guidelines is important to further improve outcomes in patients with massive PPH. Lastly, more audits like the one we performed are important to recognize and rectify any deficiencies in obstetrical practice. Dissemination of the same is pivotal to enable an open discourse on the improvement of existing obstetrical strategies.

## Abbreviations

PPH: Postpartum hemorrhage; AKUH: Aga Khan University Hospital; CS: Cesarean section; EUA: Examination under anesthesia

## Competing interests

The authors declare that they have no competing interests.

## Authors' contributions

**NN, UK and TS **were involved in data collection, statistical analysis, data interpretation and writing the manuscript. **LS **was involved in study conception and design, drafting and editing the manuscript and providing overall supervision in the project. All authors read and approved the final manuscript.

## Pre-publication history

The pre-publication history for this paper can be accessed here:

http://www.biomedcentral.com/1471-2393/11/28/prepub

## Supplementary Material

Additional file 1 **DOC Management protocol**. This is the management protocol that was introduced at Aga Khan University Hospital after the audit on massive postpartum hemorrhage conducted in 2005. Click here for file

Additional file 2 **DOC Data collection proforma**. This proforma was used to collect relevant data regarding patients with massive postpartum hemorrhage during 2008 at Aga Khan University Hospital. Click here for file
