# Supplementary material for: Evaluation of compliance and outcomes of a management protocol for massive postpartum hemorrhage at a tertiary care hospital in Pakistan
Source: BMC Pregnancy Childbirth. 2011 Apr 13;11:28. doi: 10.1186/1471-2393-11-28 (PMC3087691; doi:10.1186/1471-2393-11-28)
Supplement: Additional file 1 — DOC Management protocol. This is the management protocol that was introduced at Aga Khan University Hospital after the audit on massive postpartum hemorrhage conducted in 2005. [file 1471-2393-11-28-S1.DOC]

| **Level of care** | **Degree of**  **blood loss** | **Principal interventions** | **Additional interventions** | **Responsible person** | **Support staff** | **Timeline** |
| --- | --- | --- | --- | --- | --- | --- |
| I | Bleeding from the genital tract is 500 ml or more in the first 24 hours following delivery of the baby by vaginal or abdominal route (More than usual blood loss) | Immediately call a senior doctor i.e. Senior resident / Instructor / Consultant | Reassure patient  Ensure proper light | Person conducting the delivery | Nurse Receptionist | 10 - min |
|  |  | Confirm administration of:  Oxytocin 5-units & Ergometrine 0.4 mg as intramuscular injection at delivery of anterior shoulder of baby, or Oxytocin 10-units as intravenous injection in case of contraindication to ergometrine use | Confirm:  Controlled cord traction after delivery of baby, and  uterine massage after delivery of the placenta | Senior doctor who was called for help | Person who conducted the delivery, and  Nurse |  |
|  |  | Request laboratory to cross-match at least 2-pints of blood if not already done | Assistant to person who conducted the delivery | Nurse | Porter |  |
|  |  | Secure at least two intravenous lines with large gauge needles of # 16 or18 | Start intravenous fluids replacement with Normal Saline or Ringers Lactate | Nurse | Nursing assistant |  |
|  |  | Perform bimanual uterine-massage | Empty the urinary bladder with catheter | Senior doctor | Junior doctor  Nurse |  |
|  |  | Inspect vulva, vagina and cervix for tears | Drain vaginal haematomas and repair tears | Senior doctor | Junior doctor  Nurse |  |
|  |  | Ensure completeness of placenta | Recover placenta by controlled cord traction | Senior doctor | Junior doctor  Nurse |  |
|  |  | Initiate measurement of blood loss | Start collecting blood clots, soaked gauze-pieces, inko-pads and drape-sheets to be weighed later | Nurse | Nursing assistant |  |
|  |  | Initiate documentation | Record timings of events and interventions along with patient’s response | Nurse | Junior doctor |  |
| II | Bleeding from the genital tract is between 750 -1000 ml  (Intermediate blood loss) | Oxytocin 10-units intravenous injection boluses (repeat upto four doses within 10 minutes with intervals of 2 ½ min) | If available simultaneously give:  Misoprostol 600-mcg orally / rectally, or  ‘PG F 2α’ 5-mg intramyometrial injection  Inform patient about possibility of additional interventions to control postpartum | Senior doctor | Junior doctor  Nurse | 10 - min |
| III | Bleeding from the genital tract is between 1000 - 1500 ml  (Massive blood loss) | Balloon-Tamponade with at least 200 ml of fluid in the balloon (Two or more Foley catheter of # 24 or Special Tamponade Balloon if available) | Inform patient that additional interventions are needed now  Simultaneously commence a rapid running concentrated oxytocin infusion (with Oxytocin 40-units in Normal Saline 500ml)Consider blood transfusion as per clinical judgment of blood loss and patient status | Senior doctor / Consultant | Junior doctor  Nurse | 5 - min |
| IV | Bleeding from the genital tract is 1500 ml or more  (Near-Miss) | Commence blood transfusion as per clinical judgment of blood loss and patient status | Prepare patient to be transferred to the operating room | Nurse | Nursing assistant  Receptionist | 15 - min |
|  |  | Perform examination under general anaesthesia (EUA) in the operating room | Take consent from patient / family for possible interventions  Explore vagina, cervix and uterine cavity | Consultant | Senior/ junior doctor |  |
|  |  | Reassess for retained placenta / retained products of conception | Manually remove the placenta  Gentle curettage of uterine cavity | Consultant | Senior/ junior doctor |  |
|  |  | Reassess for vaginal haematoma | Drain haematoma | Consultant | Senior/ junior doctor |  |
|  |  | Reassess uterine atony | Uterine packing / reinsertion of Balloon Tamponade  Increase concentration of oxytocin infusion (Oxytocin 80-units in Normal Saline 500ml) | Consultant | Senior/ junior doctor  Nurse |  |
|  |  | Laparotomy for vertical and/or transverse brace-suturing of uterus (B-Lynch modifications) | Ligation of internal iliac artery (anterior division) or tying uterine vessels | Consultant | Vascular surgeon | 20 - min |
| V | Bleeding from the genital tract is uncontrolled  (Life threatening) | Subtotal abdominal hysterectomy if other measures fail | Inform patient’s family about the decision of proceeding with hysterectomy  Correction of disseminated intravascular coagulation (DIC) | Consultant | Additional Ob-consultant  Senior doctor  Junior doctor | 30 - min |
| R | Recovery Phase | Transfer patient to High dependency unit  Review patients status frequently  Seek appropriate and as needed consults  Transfer patient to routine care area only and when patient is stable | Document information given to patient or her family including the counselling done  Document whether or not any specimens were obtained and sent to the laboratoryReport incident to person incharge maternal morbidity & mortality | Senior doctor | Consultant  Junior doctor  Nurse | 24 - hr |
