# Supplementary material for: Evaluation of compliance and outcomes of a management protocol for massive postpartum hemorrhage at a tertiary care hospital in Pakistan
Source: BMC Pregnancy Childbirth. 2011 Apr 13;11:28. doi: 10.1186/1471-2393-11-28 (PMC3087691; doi:10.1186/1471-2393-11-28)
Supplement: Additional file 2 — DOC Data collection proforma. This proforma was used to collect relevant data regarding patients with massive postpartum hemorrhage during 2008 at Aga Khan University Hospital. [file 1471-2393-11-28-S2.DOC]

**Data collection proforma**

***Massive Primary Postpartum Hemorrhage during 2008***

**Basic data**

**1. Date** ______________

**2. Time** _______________

**3. Primary team** ______________________

**4. Type of delivery**_____________________________

| **Primary Management** | **Yes /No** | Point 1-10 done within 10 minutes of PPH  (Yes/No) | Senior Resident LR/Instructor and Team Leader (specify) |  |
| --- | --- | --- | --- | --- |
| 1.Call for help |  |  |  |  |
| 2.Check vitals |  |  |
| 3.2 Large bore I/V cannula |  |  |
| 4.I/V fluids N/S or hemaccel |  |  |
| 5.Cross match 2 unit & blood samples for CBC, Coag.screen |  |  |
| 6.Foley’s catheter |  |  |
| 7.Syntometrine 5 unit + 0.4  mg I/M or 10 unit Synto I/V |  |  |
| 8.Uterine massage |  |  |
| 9.Genital inspection |  |  |
| 10.Examine placenta |  |  |
| **Treatment according to cause** |  |  |  |  |
| ***Genital Trauma/Hematoma/RPOCS*** |  |  |  |  |
| EUA and proceed |  | Should be started within 15 minutes of diagnosis | Consultant |  |
| ***Uterine Atony*** |  |  |  |  |
| 10 unit synto I/V 4 doses in 10 minutes with an interval of 2 ½ minutes |  | I0 minutes for all the medical management with Oxytocics | Senior Resident LR/Instructor/ Consultant and Team Leader (specify) |  |
| Oxytocin infusion 40 unit in 500 ml N/S at 125 cc/hour |  |  |
| Misoprostol per rectal 600-800 microgram |  |  |
| Intramyometrial PGF2 Alpha 5 mg |  |  |
| Intrauterine Balloon Tamponade |  | 5 minute |  |
| EUA and proceed |  | Should be in OR in 10 minutes of balloon tamponade failure | Consultant |  |
| - Packing/Tamponade |  |  |  |  |
| - Oxytocin 80 units in 500 cc N/S |  |  |  |  |
| Laparotomy |  |  |  |  |
| - B-lynch |  |  |  |  |
| - Sequential vessel ligation |  |  |  |  |
| - Hysterectomy |  |  |  |  |
| **Decision of Transfusion of blood and blood products** |  |  | Doctor |  |
| Commencement of Blood Transfusion at 1500 ml loss |  |  | Assigned nurse |  |
| Correction of DIC |  |  | Doctor  Team leader/ Assigned nurse |  |
| **Multidisciplinary approach (if indicated )** |  |  | Senior Doctor |  |
| **Documentation** |  |  | Team leader/ Assigned nurse |  |
| **Counseling and consent process** |  |  | Senior Doctor |  |
| **Recovery Phase** |  | - Transfer to SCU - Review Patient Frequently - Consult as needed - Shift to ward once stable | Primary Team |  |

**Additional information**_____________________________________________________________________________________________________________________________________________________________________________________________________________________________________________________________________________________________________________________________________________________________________________________________________________________________________
